# Migration of metallic acupuncture threads from the breast to the right ventricle: a case report

**DOI:** 10.31744/einstein_journal/2024RC1280

**Published:** 2024-11-26

**Authors:** Nicole Simões Marini, Marcela Caetano Vilela Lauar, Mateus Galletti Oliveira, Pedro José Damato Dias Barroso, Laura Mulazzani Minuzzi Macedo, Eduardo Kaiser Ururahy Nunes Fonseca, Érica Elisângela Françolin Federicci

**Affiliations:** 1 Hospital Israelita Albert Einstein Faculdade Israelita de Ciências da Saúde Albert Einstein São Paulo SP Brazil Faculdade Israelita de Ciências da Saúde Albert Einstein, Hospital Israelita Albert Einstein, São Paulo, SP, Brazil.; 2 Hospital Israelita Albert Einstein São Paulo SP Brazil Hospital Israelita Albert Einstein, São Paulo, SP, Brazil.; 3 Universidade de São Paulo Faculdade de Medicina Hospital das Clínicas São Paulo SP Brazil Instituto do Coração, Hospital das Clínicas, Faculdade de Medicina, Universidade de São Paulo, São Paulo, SP, Brazil.

**Keywords:** Bone wires, Foreign-body migration, Foreign bodies, Breast, Mediastinum, Acupuncture techniques, Migration, Heart ventricles

## Abstract

This case report discusses the migration of a foreign body from the breast to the mediastinum, which is rarely reported, and highlights complications associated with Okibari acupuncture. This technique involves the insertion of metal fragments into the skin. A 53-year-old woman underwent mammography showing acupuncture-related metallic fragments in the breast. Cardiac evaluation revealed the presence of metallic fragments in the right ventricle and myocardium. Despite the fragments‘ location in the mediastinum, the patient remained asymptomatic, leading us to take a conservative approach. This case highlights the importance of awareness of the potential complications of foreign bodies and emphasizes the need for careful monitoring.

## INTRODUCTION

Acupuncture has been employed as a pain-relief method for centuries in Eastern medicine.^(1)^ Among the various techniques, the Okibari method involves inserting small metallic fragments beneath the skin to provide continuous stimulation for pain management. However, this technique can lead to complications, including local inflammation and fragment migration, which may result in severe conditions such as pneumothorax and cardiac tamponade.^(2–4)^

However, reports on the migration of foreign bodies to the mediastinum are limited in the medical literature. In addition to metallic wires, these foreign bodies may include Kirschner wires used for orthopedic fixation,^(5)^ breast augmentation fillers,^(6)^ and external materials used in trauma accidents.^(7)^ The outcomes of such migrations vary, and some patients require surgical intervention.

In this paper, we discuss a rare case of a foreign body migrating into the myocardium, detected using computed tomography angiography (CTA), with a probable origin in the mammary region.

## CASE REPORT

**Figure 1:**
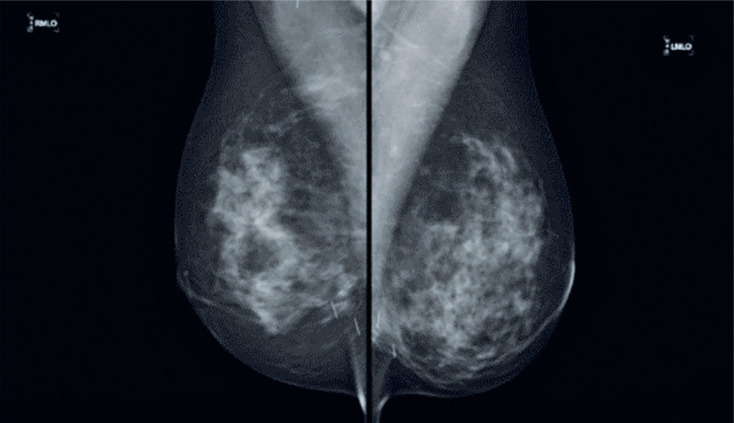
Bilateral mammography in the medio-lateral oblique view, revealing the presence of metallic linear images in the topography of the inferior quadrants

A 53-year-old female patient underwent bilateral screening mammography in 2009, which revealed linear metallic images in the lower quadrants of both breasts (Figure 1). She reported that gold threads had been inserted into her breasts during previous acupuncture sessions.

**Figure 2:**
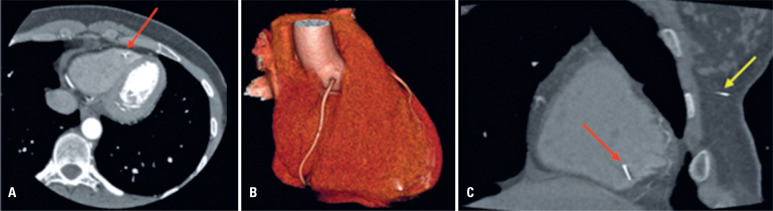
A) Computed tomography angiography (CTA) shows a metallic material in the apical region of the right ventricle (red arrow); B) CTA three-dimensional reformatting displaying the metallic fragment in the same location; C) CTA oblique plane reformatting showing that the linear metallic image within the right ventricle (red arrow) is similar to the one in the subcutaneous tissue of the left breast (yellow arrow)

**Figure 3:**
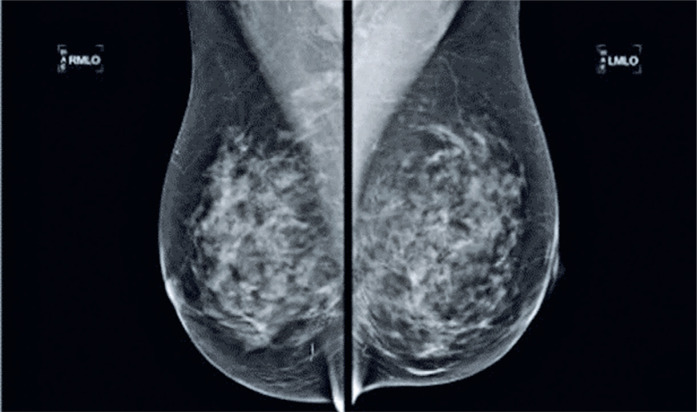
Bilateral mammography performed 1 year later, revealing the absence of one of the previously identified linear images

The following year in 2010, the patient underwent computed tomography angiography (CTA), which revealed the presence of a similar metallic material in the right ventricle (Figures 2A and B) and in the subcutaneous tissue of the left breast (Figure 2C), presumably related to acupuncture threads. Because the patient did not present with any symptoms, a conservative approach was adopted. The following year, the patient underwent another mammography scan (Figure 3), in which a metallic fragment previously observed in the right lower inner quadrant was no longer visible.

This study was approved by the Research Ethics Committee of the *Hospital Israelita Albert Einstein*, CAAE: 79124024.8.0000.0071; #6.842.011.

## DISCUSSION

The Japanese acupuncture technique for Okibari involves the insertion of metallic threads into the subcutaneous tissue, followed by cutting the proximal end beneath the skin, resulting in permanent embedding of metallic fragments in the subcutaneous layer.^(2,8)^ Various complications of this technique have been described in the literature, including the migration of metallic fragments to sites such as the medulla oblongata and urinary tract, leading to acute clinical conditions such as pneumothorax, cardiac tamponade, and cardiac embolism.^(2,9,10)^ Currently, its practice is controversial and has been discouraged by the Japanese Acupuncture Society since 1976.^(8)^

The presence of needles in breast tissue has been sparsely documented in the literature,^(4)^ with even fewer studies describing acupuncture material as a foreign body in the breast tissue. The radiological appearance of acupuncture needles is distinctive, and the presence of tiny linear metallic images, measuring up to 1.0cm, is highly suggestive of acupuncture with gold threads, especially in the presence of multiple fragments. These fragments are more commonly observed on chest radiographs within the subcutaneous tissue of the dorsal region. At our institution, this is the first case in which this appearance was observed on mammography.

Migration into the thorax can occur directly or via the venous system.^(2,8)^ In the described case, although the exact mechanism of migration could not be determined, mammographic detection of a small number of metallic fragments suggested that the myocardial foreign body migrated from the breast tissue. Incidental detection of acupuncture material in the cardiac chamber was considered serendipitous. The patient was asymptomatic; therefore, a conservative approach was chosen.

There is no consensus in the literature regarding the optimal management of such foreign body cases. Some authors argue that the potential risk of complications justifies removal through interventional procedures, whereas others advocate a conservative approach in cases of myocardial migration without tamponade.^(1,10,11)^ Furthermore, there have been some reported cases in the medical literature regarding the safe removal of foreign bodies from the mediastinum using videomediastinoscopy, which is a minimally invasive procedure. This technique has been shown to minimize complications compared to traditional thoracotomy, especially in patients who are at a higher risk of thoracotomy.^(7)^
